# Round the Hospitals

**Published:** 1916-05-27

**Authors:** 


### ROUND THE HOSPITALS.

We are indebted to the matron for calling our attention to the establishment of variations in the dietary of the staff of the Worcester Infirmary, due to war conditions. We shall be glad to have full particulars of exactly what is meant by "war conditions" and "variations in the dietary." They would seem to have been material, for the executive committee have felt called upon to express their appreciation to the staff for their acceptance of these changes and the committee's gratitude for the good spirit the staff have shown. This is the first "war conditions dietary" for a hospital staff sent us. We shall be glad to hear from other hospitals, and to know if anything is being done in this direction, and what are the estimated results not only at Worcester, but wherever war diet has become *de rigueur*. In the Institutional Worker Section in The Hospital will be found some matter of interest in this connection.

Miss Lavinia L. Dock, R.N., in charge of the Foreign Department of the *American Journal of Nursing*, in its issue for May 1916, page 751, has the following: "A Tilt in the Courts. Mrs. Fenwick, the fearless one, with her lance, the *British Journal of Nursing*, is opening a legal action with Messrs. Macmillan and their editor of the *Nursing Times*, whose names we almost fear to speak lest they look this way. We wish we could be in court when the case opens." The action referred to was tried and decided on March 14 last. A full report of the trial, published by the proprietors of The Hospital, can be procured from the publishers, 28 and 29 Southampton Street, Strand, London, W.C., price 1s.

A matron, writing to our American contemporary the *Modern Hospital*, asking for advice as to the social intercourse permitted between medical students and nurses, received the following reply: "It is the experience of veteran hospital administrators and a good many heads of training schools that it is far easier to control the relations between these young people than to forbid any intercourse between them. The best way to do this seems to be to put students and nurses on their honour, to demand their obedience to the rules of the discipline of the hospital, which should insist that while on duty their relations should be strictly restricted to business. Then, outside of duty hours, to permit certain social relations which must be controlled and guided by the matron in conjunction with the administrator of the hospital, who is responsible for the discipline of the students. The problem occurs, of course, as much here as in America; and undoubtedly the remedy lies in the same direction. If nurses and students were allowed to play tennis, hockey, and croquet together under the tolerant supervision of the home sister or assistant matron, we should hear less of midnight supper parties in ward kitchens and other breaches of hospital discipline. If they did occur, and were repeated after due warning, the offenders would qualify for expulsion.

---

It may interest many matrons, with reference to an article which appeared in THE HOSPITAL on April 22, page 87, in relation to "the National Egg Scandal," to know that *Truth* was able to announce on the 17th inst. that, thanks to the action taken by Sir Norval Helme, M.P., the Army Council, in a letter dated from the War Office on the 5th inst., states that so far as the War Office is concerned there is no objection to publication of accounts in connection with this collection, and that they have informed the Committee that this is the case. The accounts ought, therefore, to be published without a moment's further delay. Meanwhile, all who are interested in the efficient administration of movements like the Egg Collection for raising contributions from the public for charitable purposes will feel grateful to the War Office, under whose patronage the National Egg Collection has been run, for the step the Army Council has taken to secure the publication of the accounts.

---

WE beg to call attention to our correspondence column this week, in which our readers will find a suggestive letter from a hospital matron on the subject of preliminary training schools in University towns as centres of preparation for pupil-nurses. A further question of real importance is that of post-graduate training schools for nurses, to enable certificated trained nurses to attend courses of lectures, practical demonstrations, school clinics, tuberculosis dispensaries, and other active centres of special nursing work, including training in the duties of a matron, of the sisters in charge of responsible departments of a hospital, and for district, municipal, and other special work of a highly technical character.

---

A SYMPATHETIC description of the V.A.D. nurse which appeared recently in the *Yorkshire Post* deals with the relations between the V.A.D. and the professional nurse. The writer thinks that the trained nurse has nothing to fear from her untrained helper. He says: "When the war ends, in nine cases out of ten, girls will return to their homes and former occupations, a tenth, imbued with a real love for her work, will take it up seriously, spend some years at a good general hospital, and become fully qualified. We are inclined to agree that in the majority of cases this will happen. The V.A.D.s as a rule have the sense to realise that as a war hospital helper she is receiving no training in nursing. It is her relations and friends, who, quite unable to understand how circumscribed are the nursing duties which a V.A.D. nurse is called upon to perform, confuse her work with that of a trained nurse, and claim for her a skill which can only be obtained by three years spent in a well-organised training school. The V.A.D. has, we believe, no wish to enter into unfair competition with professional nurses, and medical men and women are not likely to encourage her to do so.

---

IT may truly be said that domestic economy begins with the architect. It is an open secret that the sister-matron of King's herself designed and supervised the erection of the store-rooms in the new establishment at Denmark Hill, and lapse of time has fully justified the amount of labour and brains expended in their construction. The grocery stores in particular form an object-lesson in thrift. That for the nursing and domestic staff is separate from that containing materials used in the patients' diets. Both are admirably arranged to contain goods in bulk of every variety in use, and are so planned that no articles are buried out of sight on inaccessible shelves to be forgotten. The fittings are of polished wood, and an air of spotless cleanliness and order is maintained. Every convenience for weighing and measuring is at hand. We mentally contrasted the scene with that presented by the store-rooms, much vaunted at the time, of a large Poor-Law infirmary in the vicinity of London. There a large basement had been roughly partitioned into narrow corridors, lined with open deal shelves, with transverse pieces forming gigantic pigeon-holes. A heavily bored young man was in charge, and the goods had the air of being pushed into their places merely to get them out of the way. It is quite hopeless to expect good results from a store-room unless it has been well planned from the beginning and is in charge of a capable sister, under a matron who is herself an enthusiastic housekeeper.

---

A BIG man with a shrewd, kindly face was sitting in front of the ward fire. "It's quite a treat to be out of bed for a bit, Sister." "Yes, I'm sure it must be," was the reply; "and I saw you were helping to wash up the patients' tea-things." "Indeed I did, and rinsed out the tea-cloth and hung it to dry. But for goodness' sake don't let the nurse give me away to my missis; for if she knew as I was anything of a handy man she'd expect me to act the same at home. Bless her 'eart, and I certainly draw the line at washing my own cups and saucers, much less a tea-cloth bought with my money!"

---

*** It is our invariable practice to pay for all items of news which we may publish. The minimum payment is 5s. Paragraphs of about twenty lines in length—that is to say, of 150 words or less—are preferred. In this section during the war we propose to give 10s. to the correspondent who may send us in any week the best incident connected with hospital life and work. In this way we hope to afford to all practical aid and encouragement; while those who reciprocate will be able to render us invaluable service. Contributions should be addressed to The Editor, THE HOSPITAL, 28 and 29 Southampton Street, Strand, London, W.C., and, to be in time for the current week's issue, should reach him by the first post on Mondays.

**For Matrons' and Sisters' Appointments see page 200.**

